# Taking a Breather: Advances in Interleukin 5 Inhibition for Asthma Relief

**DOI:** 10.3390/ijms231911166

**Published:** 2022-09-22

**Authors:** Oliver William Massey, Cenk Suphioglu

**Affiliations:** 1 NeuroAllergy Research Laboratory (NARL), School of Life and Environmental Sciences, Faculty of Science, Engineering and Built Environment, Deakin University, 75 Pigdons Road, Geelong, VIC 3216, Australia; 2 Institute for Mental and Physical Health and Clinical Translation (IMPACT), Deakin University, 75 Pigdons Road, Geelong, VIC 3216, Australia

**Keywords:** Interleukin-5, asthma, Mepolizumab, Benralizumab, Reslizumab, eosinophilic inflammation, immunotherapy

## Abstract

Interleukin 5 (IL-5) is a major cytokine responsible for eosinophil proliferation, migration and degranulation. Eosinophils play a considerable role in the manifestation of type 2 asthma, and therefore this makes IL-5 a unique and clinically important target for therapeutic intervention. Due to the critical role that IL-5 plays in all areas of eosinophil activity, it has been identified and targeted by three therapeutics, Mepolizumab, Benralizumab and Reslizumab. This review describes the IL-5 pathway and presents the clinical trial history of the three IL-5 inhibitors, to provide insight into the role of IL-5 in clinical asthma presentation. Additionally, this review aims to foster further investigation into the IL-5 pathway by describing current novel therapeutic discovery strategies with monoclonal antibodies.

## 1. Introduction

Over the last two decades, research into the pathophysiological mechanisms of chronic asthma has revealed the role of eosinophilic inflammation in the manifestation of symptoms. These advancements in our understanding have created a new field of immunobiological treatments targeting eosinophils for the relief of asthmatic symptoms. Interleukin 5 (IL-5) is a key mediatory signalling cytokine in eosinophil proliferation, migration and action [1,2]. Due to the narrow cellular targets of IL-5 and the key role it plays in all areas of eosinophil biology, IL-5 represents a unique area for therapeutic intervention to alleviate chronic asthma [3,4].

Patients who present with severe asthma often also present high eosinophil counts, expressing what is commonly termed the type 2 phenotype [5]. This phenotypic response is critically mediated and propagated by eosinophils, which, in turn, are influenced by IL-5 [6]. There have been two therapeutic monoclonal antibodies developed to target IL-5 to prevent eosinophilic inflammation, Mepolizumab and Reslizumab. The IL-5 receptor, IL-5Rα has also been therapeutically targeted by the monoclonal antibody Benralizumab. Whilst the efficacy of these treatments is varied, the inhibition of the IL-5 pathway has demonstrated to prevent signalling and induce cell lysis, rapidly clearing eosinophils and effectively alleviating symptoms.

This review will provide a comprehensive overview of the IL-5 signalling pathway and its relationship to asthma, whilst the second part of this review will focus on the three monoclonal antibody therapies currently approved or undergoing trials, providing a critical analysis and comparison between the three. The final part of this review will discuss immunotherapeutic discovery methods with recommendations for future therapeutic development.

## 2. IL-5 in Asthma

Eosinophils have been linked to allergy inflammation for more than 100 years, with high eosinophil numbers in airway sputum being a hallmark biomarker of T2 phenotypic asthma [7]. Eosinophils develop in the bone marrow, where they are originally CD34^+^ haematopoietic stem cells but terminally differentiated. The process of eosinophil differentiation is regulated by the GATA-1 transcription factor, which is mediated by IL-3, IL-5 and Granulocyte Macrophage Colony-Stimulating-Factor (GM-CSF) signalling. Following maturation, eosinophils migrate to the bloodstream, wherein they have a half-life of 25 h under normal homeostatic conditions [8]. When type 2 inflammatory responses are initiated, circulating eosinophils migrate from the bloodstream to the localised tissue, maturing into tissue eosinophilia [9].

T helper 2 (Th2) lymphocytes are the main IL-5 producing cells and responsible for driving eosinophilic inflammation [10]. Th2 cells release IL-5 in response to a complex signalling pathway with dendritic cells in response to allergens [11]. In addition to dendritic signalling, IL-4, another pro inflammatory cytokine, must also be present to allow for Th2 cell commitment via the activation of transcription factors STAT6 and GATA3. The IL-4 pathway is a key mechanism behind other allergic diseases, which is thoroughly discussed in the following review [12].

Due to its key role in eosinophil development, release and recruitment to tissues, IL-5 has been identified as alongside IL-4 and IL-13 as key cytokines behind the pathophysiology of T2 asthma. The close link between IL-5 and eosinophilic asthma has been clearly demonstrated through mice studies and human clinical case studies [13,14]. In asthmatic patients, the mechanisms of IL-5 take place both in the bone marrow and the airways, directly driving eosinophil differentiation and recruitment. During type 2 inflammatory reactions, eosinophils undergo a process called degranulation, where they release tissue damaging cytotoxic proteins and over 30 cytokines [15]. Eosinophils can hold around 200 of these granules and can release them progressively over time or all at once, causing a dramatic increase in inflammation. It is important to note that T2 asthma is not solely driven by allergy but can also be an immune response that is driven by innate lymphoid cells rather than IgE producing B-cells. However, eosinophils are still implicated in the pathophysiology of asthma and both allergic and non-allergic eosinophilic asthma is driven by IL-5.

## 3. IL-5 Pathway in Eosinophils

IL-5 is a 15 kDa homodimeric protein, which acts as a ligand for the IL-5 receptor complex [16]. This receptor complex is a heterodimer made of the IL-5Rα receptor chain and the βc subunit, which is also shared by IL-3 and GM-CSF [17]. The alpha IL-5 receptor chain exists in two isoforms, the transmembrane isoform and the soluble isoform. The soluble isoform is used to remove unbound IL-5 from the extracellular space, reducing ongoing activation, whereas the transmembrane isoform is required for the IL-5 signal transduction, with the cytoplasmic tail containing necessary motifs for the activation of intracellular signalling [18,19]. The regulation of the IL-5Rα is still largely unknown however, it is known that IL-5Rα expression can be influenced by cell exposure to IL-3, IL-5, IL-9 and GM-CSF [20]. Whilst playing a crucial role in the signalling of IL-5, IL-5Rα is incapable of activating intracellular signalling machinery on its own. It requires the common βc receptor subunit.

The common βc receptor chain is a crucial transmembrane protein required for IL-3, IL-5 and GM-CSF signalling [1]. The βc receptor subunit carries the needed intracellular machinery for the activation of cascading signalling pathways. Upon binding of the α receptors to their specific ligand, the βc receptor subunit is then recruited into the complex, which then cooperates with the α receptor/ligand complex to activate specific signalling pathways [21]. The universality of the common βc receptor chain is functionally analogous to the common γc receptor chain, which plays a similar role in the activation of the IL-2, IL-4, IL-7, IL-9 and IL-15 cytokine/receptor complexes [22]. The overlapping functions of these common receptor subunit chains could explain the overlapping functions that these cytokines can exert.

The formation of the IL-5/IL-5Rα/βc transmembrane complex then activates Janus Kinase (JAK) 1 and 2, which in turn causes JAK2 to phosphorylate Signal Transducer and Activator of Transcription (STAT) 1, 3 and 5, whilst JAK1 recruits signalling molecules NF-kB and PI3K [23,24,25]. Phosphorylation of STAT1, 3 and 5 causes these molecules to translocate to the nucleus, wherein they activate genes to stimulate eosinophil proliferation, such as pim-1 and cyclin D3. This is contrasted by the activation of JAK1 signal transducers phosphoinositide 3 kinase (PI3K) and Nuclear factor-κB (NF-κB), which, rather than initiating proliferation, stimulates the recruitment of eosinophils and the synthesis of pro-inflammatory cytokines [26].

IL-5 plays a pivotal role in the proliferation, migration and release of cytokines from eosinophils, therefore labelling it as a prime target for therapeutic intervention, specifically for the treatment of T2-asthma patients.

## 4. IL-5 in Clinical Intervention

**Figure 1:**
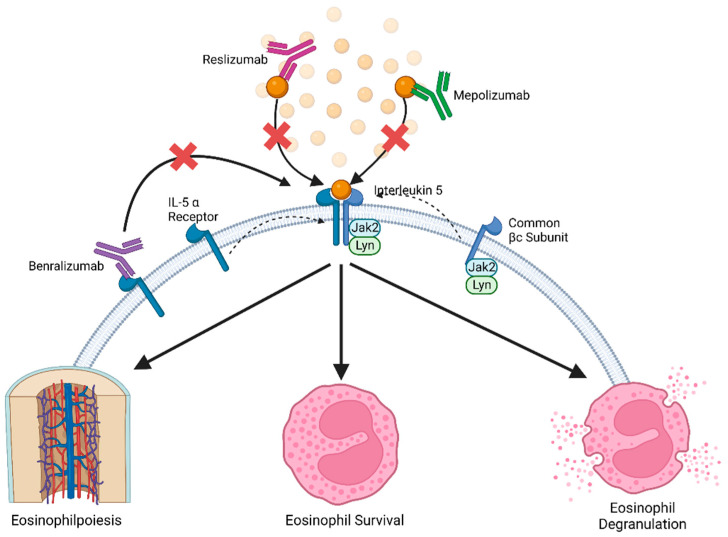
Inhibitors of the IL-5 pathway. Created with BioRender.com (accessed on 15 August 2022).

As at the time of writing of this review, three therapeutics exist that target the IL-5 pathway to neutralize its pathogenic effect. These are Mepolizumab, Benralizumab and Reslizumab and all are capable of interfering with the formation of the IL-5/IL-5Rα receptor complex. Mepolizumab and Reslizumab intervene with the IL-5 pathway by selectively binding to the IL-5 cytokine, inhibiting it from complexing with IL-5Rα (Figure 1). In contrast, Benralizumab targets the extracellular domain of the IL5Rα and upon binding, alters the receptor to be rendered inactivated (Figure 1).

## 5. Novel Antibody Discovery Methods

The therapeutics mentioned above are all monoclonal antibody based, a class of therapeutic molecules which has radically increased in recent decades. Monoclonal antibodies have been developed as highly specific treatments and are quickly becoming the leading biologic therapeutic. Alongside the increasing repertoire of therapeutic monoclonal antibodies, sophisticated discovery technologies have also been developed, which will be mentioned below. 

**Figure 2:**
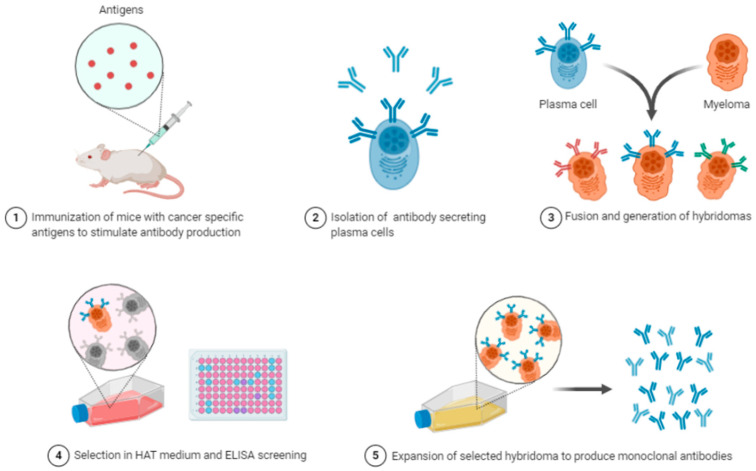
Monoclonal Antibody production via Hybridoma Technology. Reprinted from “Monoclonal Antibodies Production”, by BioRender, retrieved from https://app.biorender.com/biorender-templates/t-5f1095bb568b8300b1f30bc1-monoclonal-antibodies-production (accessed on 15 August 2022). Copyright 2022 by BioRender.

Hybridoma technologies (Figure 2) were first developed for antibody discovery, taking advantage of the native development of antibodies through influencing B-cells [27]. The ex vivo culturing of B-cells is highly demanding and difficult, hindering the identification of target specific antibodies [28,29]. Immortalization techniques for B-cells have subsequently been developed to address this issue, the first developed being hybridization. This is achieved through the fusion of primary B-cells from an antigen immunized organism with an immortal myeloma tumour cell line [30,31]. Despite being discovered almost 40 years prior to writing of this review, this technology is still common use for the discovery of novel monoclonal antibodies.

Surface display platforms are a more sophisticated class of discovery methods, which involve the generation of a library of antibodies. The strength of these surface display platforms is governed by four steps, which imitate the in vivo immune response: (i) the creation of genotypic diversity in the variable regions of the antibody, (ii) the link of genotype to the phenotype of the antibody allowing for sequencing, (iii) a selective platform which favours the binding of antibodies to target antigens, and (iv) the ability to amplify and expand the selected clones and mutants [32]. The first of these surface display technologies date back nearly 30 years, to the creation of bacteriophage display and panning. This technique was developed by George P. Smith, who discovered that bacteriophage proteins located on the outer coat could be modified to express recombinant peptides with the consequent genotype enclosed within the phage [33]. Phage display utilising the M13 bacteriophage has been used extensively. The phage display system is unique as the genotypic sequence for the surface antibody is encoded within the phage DNA, allowing for sequencing following amplification [34]. This technique was quickly adapted toward antibody screening and has become widely used since.

**Figure 3:**
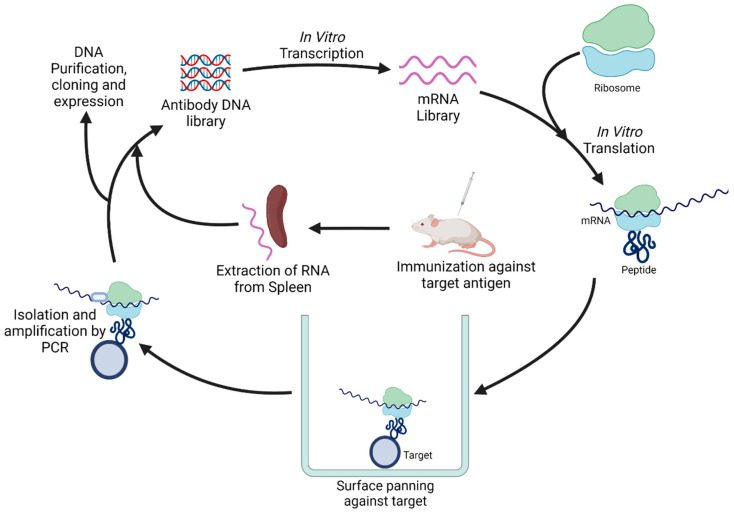
Ribosome display technology. Created with BioRender.com (accessed on 15 August 2022).

Antibodies are natively used in eukaryotic immune responses therefore a eukaryotic expression system for antibody discovery offers specific advantages over other systems. Another dominant surface display system that makes use of a eukaryotic display system is yeast display where a budding yeast, *Saccharomyces cerevisiae*, is used in screening for antibodies [35]. Akin to the concepts of phage display, yeast display sees antibody libraries being bound to yest cells via fusion with the N terminus of the Aga2p subunit [36]. In vitro surface display technology holds a specific advantage over ex vivo technologies, that is the incorporation of protein expression tags allowing for the selection of correctly assembled antibodies [37]. Another advantage specific to cell-based display technologies is the ability to use flow cytometry to analyse each cell independently. This allows for real time analysis of the display level of antibodies under controlled conditions and the quick sorting of cell populations based on their displayed molecules through high-throughput fluorescence activated cell sorting [38].An alternative in vitro display technology is ribosome display (Figure 3). This method holds the highest diversity potential (10^12^–10^13^) and holds some unique advantages over other cell display methods. Ribosome display utilises peptide sequences that lack a STOP codon, thereby leaving the polypeptide attached to the ribosome structure forming an antibody-ribosome complex. These complexes can then be panned against targets, and only requires PCR for amplification, circumventing the need for cellular transformation. Removing the need for cell culture to amplify panning product makes ribosome display a cheaper and more efficient procedure [39].

## 6. Mepolizumab

**Figure 4:**
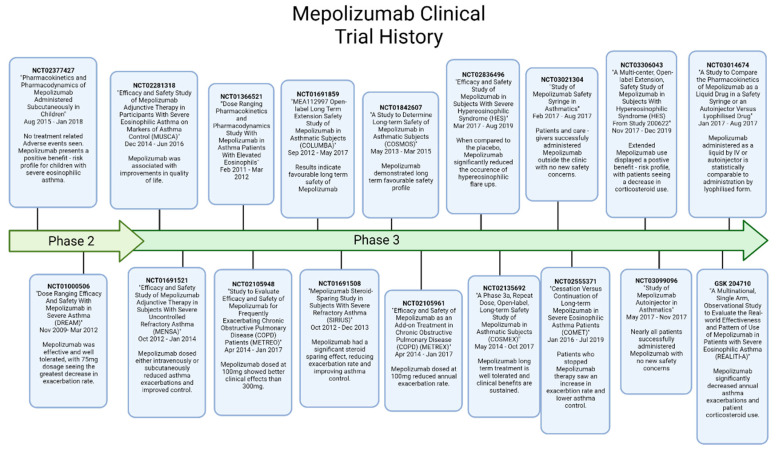
Clinical trial history of Mepolizumab. Created with BioRender.com (accessed on 15 August 2022) [49,50,51,52,53,54,55,56,57,58,59,60,61,62,63,64,65].

Mepolizumab (R03DX09) is a humanized IgG1 type antibody (Figure 4) targeted towards IL-5 and was the first anti-IL-5 treatment approved for patients with severe eosinophilic asthma [40]. Being targeted to the IL-5 cytokine, Mepolizumab binds to IL-5, preventing its interaction with the IL-5 alpha receptor chain, effectively inhibiting the pathway [41]. Due to the key role IL-5 plays in stimulating eosinophil proliferation, migration and synthesis of pro-inflammatory molecules, inhibiting the IL-5 pathway cripples their function, leading to rapid clearance and lowering population counts [42,43]. Following its approval in 2015 for the treatment of eosinophilic asthma [40], Mepolizumab has also been approved as a treatment for eosinophilic granulomatosis with polyangiitis and for hypereosinophilic syndrome [44,45]. Despite its recent success, the development of Mepolizumab has been turbulent. Whilst Mepolizumab effectively reduce asthma biomarkers, trials conducted in 2000 and 2007 were considered unsuccessful as to the lack of clinical efficacy [46,47]. In this point of development for Mepolizumab, the lack of clinical efficacy was deemed a result of the patient groups, as patients had been selected for the trial based on clinical and physiological characteristics, as opposed to eosinophil counts.

Following these revelatory studies, a larger scale clinical trial was conducted to identify the target population that would benefit from Mepolizumab. The DREAM (Dose Ranging Efficacy And safety with Mepolizumab; NCT01000506) trial was conducted in 2012. The DREAM trial demonstrated Mepolizumab was effective in patients presenting with severe asthma, high eosinophil inflammation and a history of exacerbations. Importantly, DREAM also presented a 54% reduction in exacerbation frequency granting higher quality of life. Following DREAM, a phase III clinical trial termed MENSA (MEpolizumab as adjunctive therapy iN patients with Severe Asthma; NCT01691521) displayed direct relationship between the reduction in eosinophil counts and exacerbation rate. The phase III MENSA trial also demonstrated a 53% reduction in exacerbation rates, increasing patient quality of life. This direct relationship was further demonstrated in the MUSCA (Mepolizumab adjUnctive therapy in subjects with Severe eosinophiliC Asthma; NCT02281318) and the SIRIUS (SteroId Reduction with mepolizUmab Study; NCT01691508). The SIRIUS trial also demonstrated a reduced corticosteroid dosage in patients resulted in no increase in exacerbations when combined with mepolizumab. 

The final study conducted prior to the approval of Mepolizumab as a combined treatment was REALITI-A (REAL world effectiveness of mepolizumab in paTIent care–Asthma; NCT03562195). The REALITI-A study possessed a large population of 368 patients. This study displayed Mepolizumab significantly decreased annual asthma exacerbations and reduced patient reliance on corticosteroids. Additionally, no new safety concerns were presented.

Mepolizumab, whose commercial name is “Nucala”, is delivered by subcutaneous injection with the recommended dose of 100 mg every 4 weeks [48]. The dosage of Mepolizumab is high in comparison to the following therapies, however being delivered subcutaneously it may be more appealing to certain patients than the following therapeutics.

## 7. Reslizumab

**Figure 5:**
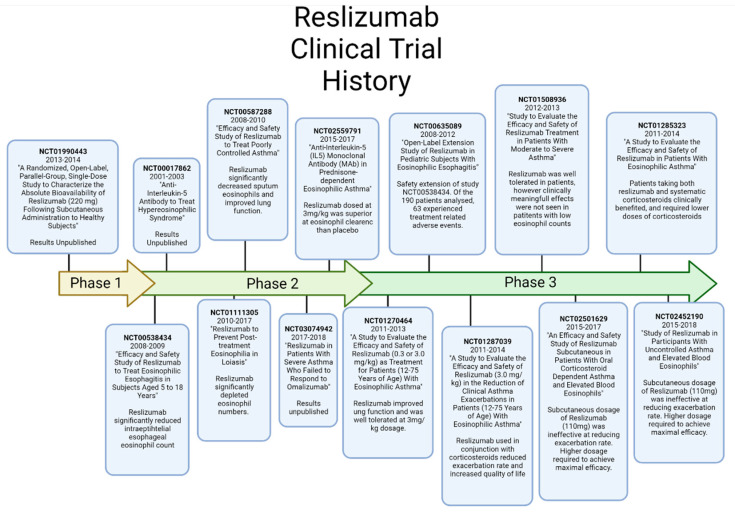
Timeline Summary of Reslizumab Clinical Trials. Created with BioRender.com (accessed on 15 August 2022) [66,67,70,71,72,73,74,75].

Reslizumab (R03DX08) is a humanized monoclonal antibody targeting IL-5 directly (Figure 5). Reslizumab has been indicated as an add-on treatment for asthma, to be used in conjunction to corticosteroids. Reslizumab has a remarkably high binding affinity for IL-5 where it prevents binding to the IL-5Rα by occupying the binding site. By binding to IL-5, theoretically this should prevent eosinophil proliferation and activity, however this is yet to be conclusively determined.

To date, there have been six completed clinical trials involving Reslizumab for the treatment of asthma, with each having their own findings. The earliest trials of Reslizumab found it was highly effective at clearing blood eosinophils for up to 16 weeks, however there was no significant effect on asthma symptoms [46]. Following this initial phase I trial, further studies into Reslizumab was conducted in 2011. This phase II trial was a double-blind placebo study with 106 patients randomised to Reslizumab at 3 mg/kg or a placebo. This trial ran over 15 weeks and found Reslizumab had a statistically significant therapeutic effect in patients presenting with elevated eosinophil counts [66]. In this study, Reslizumab significantly improved lung function, however had no significant effect on asthma control. Following these results, two phase III trials were performed by the same researchers in 2015, where Reslizumab was investigated in another double blind, placebo controlled, randomised study design, however the population in this study was vastly increased. Across both studies, 477 patients received Reslizumab in combination to their current asthma treatments. This study found significant improvements in asthma exacerbation rate and lung function when used with patients prescribed with oral corticosteroids and blood eosinophil counts higher than 400 cells/μL [67]. Following these promising results from the two previous trials, two more phase 3 trials were conducted in 2016 termed the ANDHI and OSTRO trials. The most recent two trials for Reslizumab looked into its efficacy for asthma and the reduction in chronic rhinosinusitis. The ANDHI trial investigated the effect of Reslizumab in 656 patients who suffered from uncontrolled eosinophilic asthma focusing on exacerbation rate, lung function and associated nasal polyposis symptoms. This trial found that patients receiving 30 mg of Reslizumab every 8 weeks reduced their risk of asthmatic exacerbation by 49%, with significant improvements in lung function recorded [68].

Reslizumab, also known as Cinqair/Cinqaero is delivered to patients intravenously over 20–50, at a dosage of 0.3 mg for every kg of body weight. It is recommended the for continued effect, Reslizumab must be administered every 4 weeks [69]. Whilst possessing the lowest required dose, Reslizumab is required to be administered over 20–30 min, whereas Mepolizumab and the following therapeutic, Benralizumab, have no such requirement.

## 8. Benralizumab

**Figure 6:**
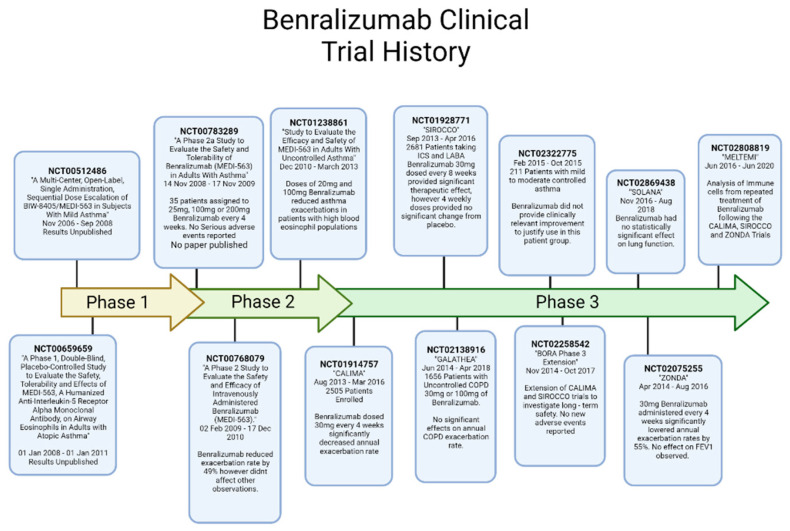
Timeline Summary of Benralizumab Clinical Trials.Created with BioRender.com (accessed on 15 August 2022) [76,81,82,83,84,85,86].

Benralizumab is an IgG1 monoclonal antibody, which targets the IL-5Rα protein (Figure 6). This monoclonal antibody is approved for use in the US, Europe, Japan, Canada and Australia as an add-on therapy for the treatment of eosinophilic asthma. Benralizumab has a rich history of clinical trials spanning from 2007 to the current day. The initial phase 1 trials for Benralizumab were run between 2007 and 2008 and 2008 and 2012 for smaller populations, however the results for these trials are not publicly available. There have been four completed phase 2 trials to date, three of which have available results. The earliest phase 2 trial ran from 2008 to 2009 and the safety of Benralizumab in 25 individuals, with treatment emerging serious adverse effects recorded. Following the aforementioned safety trial, two more phase 2 trials were conducted with much higher populations and looking into the efficacy of Benralizumab for uncontrolled asthma and chronic obstructive pulmonary disease. The first of these two studies, titled “Study to Evaluate the Efficacy and Safety of MEDI-563 in Adults with Uncontrolled Asthma”, possessed a population of 609 patients randomized to placebo or Benralizumab. This study found no safety issues with Benralizumab, with doses of 20 mg and 200 mg providing a reduction in asthma exacerbations within individuals who possess blood eosinophil baseline counts of 300 cells/μL or higher [76]. The second of these studies, titled “A Study to Evaluate the Effectiveness of MEDI-563 in Subjects with Chronic Obstructive Pulmonary Disease (COPD)” had a smaller population of 101 patients with 3% or higher baseline blood eosinophil counts. This study found no significant effect of Benralizumab on COPD exacerbations; however, it warranted further study into patients displaying the eosinophilic phenotype [77].

As at the time of writing this review article, there have been 16 phase 3 clinical trials involving Benralizumab, investigating its safety in larger populations but also its interactions and efficacy with other treatments. For this review, we will focus on the three most influential trials of Benralizumab, which has led to its approval, namely the CALIMA, SIROCCO and ZONDA trials which solidified its efficacy as an add-on therapy.

The CALIMA trial was conducted over 56 weeks between 2013 and 2016. Of the 2508 patients enrolled, 866 were dosed with 30 mg of Benralizumab either every 4 weeks or every 8 weeks, with the remaining patients being administered a placebo. Within this trial, Benralizumab significantly reduced the annual rate of asthma exacerbations up to 36%, supporting the results from the SIROCCO trial, which occurred beside CALIMA [78]. In addition to the reduction in annual asthma exacerbations, Benralizumab demonstrated improved FEV1 within patients throughout the course of treatment, however no improvement in FEV1 was observed in patients whose base eosinophil count was below 300 cells/μL.

The SIROCCO trial was conducted between 2013 and 2016, involving 2681 patients across 17 countries. Unlike previous trials, which investigated Benralizumab only, this study looked into its efficacy in patients whilst taking high dose of inhaled corticosteroids and long-acting β_2_ agonists, who still presented with uncontrolled asthma [79]. During this study, Benralizumab significantly reduced patient’s annual exacerbation rates by a total of 51% after 48 weeks of continuous treatment. In addition, Benralizumab significantly improved lung function, increasing FEV1 when compared with the placebo treatment. Whilst these results are promising, it is important to note that these significant improvements were observed within patients who had blood eosinophil counts equal to or above 300 cells/μL. In addition, the greatest improvement in symptoms was observed within patients who underwent treatment every 8 weeks, as opposed to every 4 weeks.

Benralizmab, whose commercial name is “Fasenra” is administered intravenously by syringe at a dosage of 30 mg for every 4 weeks, then every 8 weeks after three doses [80]. No adverse effects have been reported for doses up to 200 mg. Being an intravenous injection, Benralizumab may appear more appealing to patients over the 20–50 min administration of Reslizumab however the 30 mg dosage is untied to body weight, thereby creating a higher dose for some individuals.

## 9. Conclusions

Over the last two decades, there have been great strides in the development of biologics for asthma. The IL-5 pathway represents a unique opportunity for therapeutic intervention, allowing for the rapid clearance of eosinophils. The clearance of eosinophils from the airways prevents the release of inflammatory mediators and granule specific proteins, namely major basic protein (MBP), cytokines and lipid based inflammatory mediators. These molecules cause airway remodelling and airway hyperresponsiveness, which are hallmark symptoms of asthma. By preventing the release of these molecules through the clearance of eosinophils, asthma symptoms can be alleviated. Three therapeutics have been developed so far, targeting this pathway for the treatment of asthma, with their trials providing valuable insight into the mechanics behind eosinophilic asthma. These trials have proved the therapeutic efficacy of anti-IL-5 and anti-IL-5Rα antibodies by reducing annual asthma exacerbations. It is important to note however, that these anti-IL-5 treatments are not a sole solution to asthma, rather they have been approved as an add-on therapy for patients already taking high dosage corticosteroids. Additionally, there are other cytokines which could be targeted for asthma, namely IL-4 and IL-13 and whilst there has been some study in this area, only one IL-4 inhibitor has been approved for asthma treatment (Dupilumab). Therefore, there remains scope for further research targeting the IL-5 pathway for asthma therapeutics.

## Data Availability

Not applicable.
